# The effects of semantic similarity on Mandarin speakers’ referential expressions

**DOI:** 10.1177/17470218231154578

**Published:** 2023-03-01

**Authors:** Yangzi Zhou, Holly P Branigan, Yue Yu, Martin J Pickering

**Affiliations:** Department of Psychology, The University of Edinburgh, Edinburgh, UK

**Keywords:** Language production, sentence production, similarity-based interference, reference, referential expression, Chinese

## Abstract

Previous research has found apparently contradictory effects of a semantically similar competitor on how people refer to previously mentioned entities. To address this issue, we conducted two picture-description experiments in spoken Mandarin. In Experiment 1, participants saw pictures and heard sentences referring to both the target referent and a competitor, and then described actions involving only the target referent. They produced fewer omissions and more repeated noun phrases when the competitor was semantically similar to the target referent than otherwise. In Experiment 2, participants saw introductory pictures and heard sentences referring to only the target referent, and then described actions involving both the target referent and a competitor. They produced more omissions and fewer pronouns when the competitor was semantically similar to the target referent than otherwise. We interpret the results in terms of the representation of discourse entities and the stages of language production.

## Introduction

Speakers often wish to refer to an entity that has been previously mentioned. To do so, they must retrieve a referent from memory and linguistically encode it. But they often have several choices about what to do. For instance, after saying *Tom kissed a girl*, speakers can explicitly refer to Tom with the repeated noun phrase (NP) *Tom* (e.g., *Tom was very happy*). Alternatively, they can use a less explicit expression such as the pronoun *He (*e.g., *He was very happy*). Moreover, speakers of languages such as Mandarin can be even less explicit by omitting the subject altogether, as the context makes its identity clear (e.g., *很高兴, /hen3 gao1xing4/*, “was very happy”).

But the process of encoding a referential expression is not straightforward. Accounts of language production assume that speakers first conceptualise what they want to say in relation to its context—specifically, its discourse context when the utterance follows previous utterances. Next, they formulate by converting this representation into linguistic representations concerned with grammar and sound, at which point they draw on lexical entries (lemmas and word forms). Finally, they articulate their utterance, typically by speaking (Levelt, 1989). Language production involves competition between lexical representations (Levelt et al., 1999), and sometimes the utterance can involve two entities that are similar in some respects (e.g., both are animate). How exactly does the similarity between entities influence the way in which speakers retrieve the concept of the referent from memory (during conceptualisation) and linguistically encode it (during formulation)? And how do these processes influence speakers’ referential behaviour? In this article, we contrast two apparently conflicting claims about how semantic similarity might influence speakers’ use of referential expressions and report two experiments in Mandarin.

Semantic similarity affects the production of single words, as demonstrated by the picture–word interference paradigm (Lupker, 1979; Schriefers et al., 1990). In that paradigm, speakers name a target picture while disregarding a visually or auditorily presented competitor word. Naming latencies for the target (e.g., a frog) tend to be longer when the competitor word is semantically similar to the target (e.g., *fish*) than when the competitor word is semantically dissimilar to the target (e.g., *chair*). This finding suggests that speakers undergo interference when accessing lexical information associated with the target in the presence of a semantically similar entity.

To explain such effects of similarity on the production of single words, Roelofs’ (1992) lexical access model suggested that the target lemma (i.e., the syntactic component of a lexical entry) and the competitor lemma undergo greater lexical competition when they are semantically related than otherwise. Activation of the target lemma is delayed under these circumstances because the lemma of the semantically similar competitor is also activated via shared conceptual nodes (e.g., ANIMAL), leading to interference in production. Such interference does not occur when the words are semantically unrelated.

But does similarity also affect referential processing? In the picture–word interference paradigm discussed above, participants simply name a target stimulus presented simultaneously with a competitor word (or within a very short window, usually less than 200 ms). In other words, speakers do not need to temporarily remember the information associated with the target and competitor entities and subsequently retrieve that information. But the situation is different when speakers refer to an already-introduced entity. They must have encoded information about the referent and must retrieve that information when they plan to refer to it. So how is this retrieval process affected if they have also previously encoded a semantically similar “competitor” referent?

Many studies have shown the effects of semantic similarity on speakers’ reference production. In some studies, participants freely provide continuations to target sentences involving either two similar or two dissimilar entities (Fukumura & van Gompel, 2011). In other studies, they describe target pictures containing either two similar or two dissimilar entities (e.g., Gennari et al., 2012). Studies using both paradigms have found that speakers’ referential behaviour varies according to whether the context contains another potential referent that is similar to the target referent or dissimilar to it. But the specific consequences of such similarity on referential behaviour remain controversial, in part because studies have shown apparently conflicting patterns of results. As we now discuss, some studies suggest that similarity enhances explicitness, whereas others suggest that it reduces explicitness.

### Similarity enhances explicitness

Some studies have found that greater semantic similarity leads to more explicit referential expressions. All these studies have used languages in which the key choice of referring expression is between the repeated NP, which is more explicit, and the pronoun, which is less explicit. They find that when the target referent is similar to the competitor, speakers tend to use more explicit expressions such as repeated NPs, and fewer less explicit expressions such as pronouns, to refer to the target referent (Arnold & Griffin, 2007; Fukumura et al., 2011, 2013).

A well-studied dimension of similarity is gender—same-gender entities share a semantic feature that different-gender entities do not. In several studies in English and other languages such as French, speakers tended to use repeated NPs and fewer pronouns to refer to an entity when it was the same gender as its competitor than when it was a different gender (the *gender congruency effect*; e.g., Arnold et al., 2000; Arnold & Griffin, 2007; Fukumura et al., 2010, 2011, 2022).

The gender congruency effect has traditionally been attributed to referential ambiguity avoidance (e.g., Arnold et al., 2000; Karmiloff-Smith, 1985). That is, when the target referent and a competitor have the same gender (e.g., Tom and John), a gendered pronoun is ambiguous (*he* can refer to either Tom or John). Therefore, speakers are more likely to use a repeated NP (e.g., *Tom*) than when the target referent and its competitor have different genders (e.g., Tom and Mary) and so a gendered pronoun is unambiguous (*he* can only refer to Tom). The ambiguity avoidance account assumes that speakers design their utterances by taking into account their addressee’s needs (audience design; Clark & Murphy, 1982). In other words, speakers select an explicit referential expression because it avoids referential ambiguity, thus enabling the addressee to uniquely identify the referent.

However, speakers may choose more explicit referential expressions for reasons other than ambiguity avoidance. Arnold and Griffin (2007) had participants describe a story involving either one character (Mickey) or two characters (both the target referent Mickey and a different-gender competitor Minnie). Speakers produced more repeated NPs and fewer pronouns in the two- than the one-character condition. In both conditions, a pronoun (*he*) is unambiguous, and so these results could not be due to ambiguity avoidance. Instead, Arnold and Griffin argued that the results were due to the presence of another entity in the discourse representation (which means that the effects occurred during conceptualisation). They suggested that the competitor referent competed with the target referent for attentional resources, rendering the target referent less active (hence less accessible) than when no such competitor was present. As a consequence, participants used a more explicit referential expression. Arnold and Griffin noted that their results were consistent with a competition-based account of the gender congruency effect, in which entities that share semantic features compete for attention, with the strength of competition reflecting the degree of overlap. However, their experiments did not manipulate the degree of semantic similarity between the two entities, and it is possible that the effects would occur with any two entities, irrespective of semantic similarity.

Fukumura et al. (2011) studied the effects of semantic similarity in a different manner: by manipulating the *situational similarity* between two entities. Participants described pictures involving a target referent and a competitor who were either in the same situation (e.g., both were on horses, and therefore they were semantically more similar) or in different situations (e.g., the target referent was on a horse but the competitor was standing, and therefore they were semantically less similar). Participants used fewer pronouns and more repeated NPs in the same-situation condition than in the different-situation condition (see also Fukumura et al., 2013, for a similar study in Finnish). It is worth noting that in Fukumura et al. (2011, 2013) both entities were included in the action picture, but only the target referent was involved in the target event. This might lead to some referential ambiguity caused by the presence of the competitor when speakers were describing the action of the target referent.

Fukumura et al. (2011) explained these effects from a discourse processing perspective, in which the choice of referential expression depends on the activation that the referent receives from associated semantic feature nodes (e.g., for *on a horse*)—thus, during the process of conceptualisation. The presence of a similar competitor reduces the amount of activation directed to the target referent, reducing its accessibility in the discourse model. Speakers must consequently activate more information about the target referent to successfully retrieve it from memory, making them select an explicit referential expression.

This account is consistent with the claim that speakers tend to use less explicit referential expressions for more accessible referents, and more explicit referential expressions for less accessible referents (Ariel, 1990; Chafe, 1976; Givón, 1983; Grosz et al., 1995; Gundel et al., 1993). This claim has been confirmed by studies manipulating various factors that affect accessibility. For example, pronouns are more frequently used when the antecedent expression corresponding to the referent is the syntactic subject (Arnold, 2001; Fukumura & van Gompel, 2010; Stevenson et al., 1994), is first-mentioned (Gernsbacher, 1989; Gernsbacher & Hargreaves, 1988), is recently mentioned (Ariel, 1990; Givón, 1983), is long (Karimi et al., 2014), or is animate rather than inanimate (Fukumura & van Gompel, 2011). In all cases, pronoun use is associated with a more accessible antecedent (as when it is semantically dissimilar to the competitor), and full NPs with a less accessible antecedent (as when it is semantically similar to the competitor). One possibility is that accessibility (operationalised as relative level of activation) determines the type of referential expression that speakers consider during planning reference.

### Similarity reduces explicitness

Strikingly, another group of studies has suggested that semantic similarity can have the opposite consequence. In other words, greater similarity can lead to *less* explicit referential behaviour. Gennari et al. (2012) presented English, Serbian, and Spanish participants with scenes involving an animate agent (e.g., a woman) who acted on either an animate patient (e.g., a man) or an inanimate patient (e.g., a sandbag). Participants answered questions about the animate patient (e.g., *Who is bald?*) or the inanimate patient (e.g., *What’s orange?*) which prompted relative clause responses. These responses could involve an active structure (*The man/sand bag that the woman is punching*), a passive structure mentioning the agent (*The man/sand bag that is punched by the woman)*, or a passive structure omitting the agent (*The man/sand bag that is punched)*.

In all three languages, participants showed a tendency (to varying degrees) to produce passive relative clauses (e.g., *The man that is being punched [by the woman]*) when the agent and the patient were both animate than when the agent was animate and the patient was inanimate. Most relevantly, they were more likely to omit the agent when the patient was animate (and therefore had the same animacy as the agent) than when the patient was inanimate (and therefore had a different animacy from the agent). In other words, they were more likely to say *The man that is being punched* than *The sand bag that is being punched*. Thus, speakers tended to omit an optional agent when it had the same animacy as the patient (or in theory, when the patient was animate, as only the animacy of patient was manipulated in Gennari et al. (2012), but the agent was always animate). So semantic similarity led to a less explicit referential expression (omission)—the opposite pattern of results from Fukumura et al. (2011, 2013). In other words, Gennari and colleagues contrasted full NP versus omission, whereas Fukumura and colleagues contrasted full NP versus pronoun.

Using active transitive sentences in Mandarin, Hsiao et al. (2014) found effects that were compatible with Gennari et al. (2012). Participants described scenes in which an animate agent (e.g., an old gentleman) performed an action (e.g., kicking) on an animate patient (e.g., a robber) or an inanimate patient (e.g., a football). Consistent with Gennari et al. (2012), participants were more likely not to express the agent (i.e., the subject) when the patient was also animate (e.g., kicked the robber) than when it was inanimate (e.g., kicked the football).

Gennari et al. (2012) explained their effects by arguing that speakers’ referential behaviour is affected by the ease of processing during grammatical encoding (a stage of formulation). They proposed that the presence of a semantically similar competitor causes interference during lexical retrieval and grammatical function assignment, resulting in lexical inhibition (e.g., of the lexical entry associated with the agent). Thus, their explanation is in terms of linguistic processing rather than discourse processing. To avoid disfluency, speakers choose alternatives that allow them to maintain production efficiency, including omitting one entity altogether.

In sum, previous studies have found apparently conflicting effects of semantic similarity on speakers’ referential behaviour. Some studies show that when the discourse contains two semantically similar entities, speakers tend to be *more* referentially explicit (e.g., more likely to produce repeated NPs; Fukumura et al., 2011, 2013), whereas other studies show that they tend to be *less* referentially explicit (e.g., more likely to omit entities; Gennari et al., 2012; Hsiao et al., 2014). And theoretical accounts of semantic similarity effects accordingly make apparently contradictory predictions, on one hand proposing that semantic similarity leads speakers to activate more information during referential processing in a way that facilitates retrieval and causes speakers to be referentially more explicit, and on the other hand proposing that semantic similarity leads speakers to activate less information during referential processing that interferes with retrieval and causes speakers to be referentially less explicit.

## The present study: resolving the different findings

So, what is the nature of semantic similarity effects on the speaker's referential behaviour, and how do these effects arise during language production? Previous findings do not appear consistent with each other, but it is difficult to draw strong conclusions because the studies differ along two potentially important dimensions. First, they differ in production conditions—whether both the target and competitor entities needed to be described in the target event. In studies showing that similarity enhances explicitness (e.g., Fukumura et al., 2011, 2013), both the target entity and the competitor were introduced in the preceding discourse (e.g., a man standing and a man on a horse), but only the target was involved in the target event (e.g., a man getting off a horse). Thus, participants had to distinguish between two entities in their discourse model, but during production, they had to retrieve and encode only the target referent.

In contrast, in studies showing that similarity reduces explicitness, either only the target entity (but not the competitor) was introduced in the preceding discourse (Hsiao et al., 2014), or neither was introduced (Gennari et al., 2012), but both entities were involved in the target event (e.g., a woman kicking a man, as in Hsiao et al., 2014). Under these conditions, participants did not need to distinguish between more than one entity in their discourse model. But during production, they had to retrieve and encode information associated with both entities. This was an especially difficult process because the competitor was not salient (i.e., not established) in the discourse representation, and so participants had to make an additional effort to retrieve it.

Second, the studies differed in their dependent variables—participants’ choice of referential expressions. Studies revealing that similarity enhances explicitness measured the use of repeated NPs versus pronouns in different similarity conditions (e.g., Fukumura et al., 2011, 2013), whereas studies revealing that similarity reduces explicitness measured whether an argument was mentioned or omitted by contrasting the use of omissions versus pronouns (e.g., Gennari et al., 2012; Hsiao et al., 2014). This difference could be due to the nature of the languages or the structures investigated in each type of study. In the former studies, omission was not possible (as neither English nor Finnish allows subject omission). In the latter, omission of an argument was possible (as English permits agent omission in passive relative clauses, and Mandarin permits subject omission). Therefore, the contrasting results could reflect the differences in permissible referential expressions between the two sets of studies. However, none of the studies looked at all three types of referential expression. Thus, the current study enables us to go beyond previous research and investigates the generalisation of the semantic similarity effects to pro-drop languages. In our experiments, the repeated NP is the most explicit referring expression, and omission is the least explicit. Unlike previous studies, the pronoun is not the least explicit possibility.

To resolve the nature of semantic similarity effects on referential behaviour, and how these effects arise during production, we conducted two picture-description experiments in Mandarin. We used Mandarin because it allowed three types of response that vary in explicitness: repeated NPs, pronouns, and argument omission (note that Mandarin allows omission of the grammatical subject as well as object arguments). In both experiments, participants heard an introductory discourse and then described a target event that involved a target referent. In Experiment 1, the introductory discourse mentioned the target referent and a competitor, but the target event involved only the target referent, similar to Fukumura et al. (2011, 2013). In Experiment 2, the introductory discourse mentioned only the target referent, but the target event involved both the target referent and a competitor, similar to Hsiao et al. (2014).

We manipulated the semantic similarity between the target referent and the competitor through a novel semantic dimension: occupational similarity. In particular, the competitor was either occupationally similar (e.g., *杀人犯, /sha1ren2fan4/*, “murderer” in the high-similarity condition) or occupationally dissimilar (e.g., *校对员, /jiao4dui4yuan2/*, “proofreader” in the low-similarity condition) to the target referent (e.g., *杀手, /sha1shou3/*, “killer”). Both entities were animate and had the same gender. Note that the same pronoun */ta/* is used for both female and male entities in spoken Mandarin, so that pronouns are referentially ambiguous, regardless of whether or not the target referent and the competitor have the same gender. We measured whether participants referred to the target entity using a repeated NP, a pronoun, or did not refer to it overtly (i.e., an omission).

In this way, we can determine the cause of the contrasting findings in the previous studies. If they are due to differences in the choice of referential expressions (i.e., the second explanation above) and crucially whether omission was possible or not, then both Experiments 1 and 2 should pattern with previous studies in which omission was one of the referential options, namely those studies that find similarity reduces explicitness (e.g., Gennari et al., 2012; Hsiao et al., 2014).

But if Experiment 1 finds that similarity enhances explicitness and Experiment 2 finds that similarity reduces explicitness (with the choice of referential expression being the same), then the explanation must be due to another difference between the studies—most obviously, that only the target needed to be described in the target event in Experiment 1 but both the target and competitor entities needed to be described in the target event in Experiment 2 (i.e., the first explanation above). We now report these experiments before returning to interpret their findings.

## Pre-test

To validate the degree of semantic similarity (high vs. low) in the target–competitor pairs, we first conducted a similarity rating pre-test in which 20 native Mandarin speakers from the same population as the main experiment participated in exchange for ¥5. We constructed 35 pairs of entities, each comprising two occupational names that we judged to be semantically similar (e.g., *杀手, /sha1shou3/*, “killer” and *杀人犯, /sha1ren2fan4/*, “murderer”) or dissimilar (e.g., *杀手, /sha1shou3/*, “killer” and *校对员, /jiao4dui4yuan2/*, “proofreader”). None of the target–competitor pairs were associatively related; note that we selected pairs for which our intuition was that the relationship was semantic rather than purely associative, as picture–word interference studies (e.g., Alario et al., 2000) have found facilitatory effects of associative relations rather than the inhibitory effects that are found for semantic relations (e.g., Schriefers et al., 1990).

We carried out the pre-test using the Chinese online survey tool WJX (https://www.wjx.cn/). We created two lists using a Latin Square design so that participants saw either the similar or the dissimilar pair from each item set: list A had 17 experimental items from the high-similarity condition and 18 from the low-similarity condition, and list B had the opposite distribution. Participants were asked to judge how semantically or conceptually similar the two concepts were using a 1–7 Likert-type scale (1 = *extremely dissimilar*, 4 = *neutral*, 7 = *extremely similar*), using guidance such as *To what extent do they play a similar role in real life; To what extent do they have a similar working environment?* to aid their judgements. They were instructed not to make their decisions based on whether the physical appearance of the characters was similar or not. The study took around 3 min to complete.

Because the dependent variable was ordinal (on a 7-point Likert-type scale), we analysed the data using cumulative link mixed models in the ordinal package (R. H. B. Christensen, 2018) of the statistical software R (the clmm function). The final model included Similarity (high- vs. low-similarity) as the fixed variable which was centred prior to analysis (Baayen, 2008), and subject and item as random variables (Baayen et al., 2008). The mean similarity rating for high-similarity pairs was 5.56 (range 4.90–6.70; *SD* = 1.00), and the mean similarity rating for low-similarity pairs was 2.12 (range 1.50–2.60; *SD* = 1.17); this difference was significant (β = 6.56, *SE* = 0.39, *z* = 16.92, *p* < .001). We thus used all 70 pairs as experimental items.

## Experiment 1

Experiment 1 had a similar logic to those reported in Fukumura et al. (2011, 2013). If semantic similarity effects were contingent on production conditions (as determined by the structure of the experiment), we expected to find results consistent with Fukumura et al. (2011, 2013). That is, speakers would be referentially more explicit when the target referent and competitor were semantically similar than when they were semantically dissimilar. Hence, they should produce more referring expressions that are more explicit and fewer referring expressions that are less explicit to refer to the target referent when the target referent and competitor were semantically similar than when they were dissimilar. But if semantic similarity effects were due to whether omission is one permissible referential expression, we expected to find results compatible with Hsiao et al. (2014). That is, speakers should produce more referring expressions that are less explicit and fewer referring expressions that are more explicit to refer to the target referent when the target referent and competitor were semantically similar than when they were dissimilar.

### Method

#### Participants

Thirty-five University of Edinburgh students (8 males; *M_age_* = 23 years) who were native speakers of Mandarin took part in exchange for £5. All participants were born in China and lived there until at least age 18 and had spent less than 2 years in the United Kingdom.

##### Items

**Figure 1.**
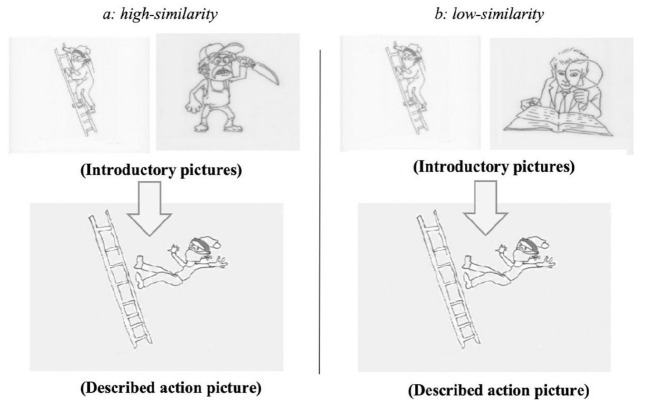
Example experimental pictures (Experiment 1): (a) high-similarity and (b) low-similarity.

We constructed 35 sets of experimental items (see the Online Supplementary Material). Each set included three introductory pictures (top panels in Figure 1a and b), one action picture (bottom panels in Figure 1a and b), and two introductory sentences (such as 1a and b).

**(1a). *High-similarity*:**

有一位心狠手辣杀人不眨眼的杀手，站在一名杀人犯旁边，正在顺着梯子逃离作案现场。

突然，_________________。

You yiweixinhenshoula sha renbuzha yande shashou, zhan-zaiyiming

Have one.CL ruthlesskill people noblink eye DE killer, stand-ZAI one.CL

sharenfan pangbian, zheng-zai shun-zhetizitaolizuoanxianchang.

murderer beside, ASPalong-ZHE ladder escape commit.a.crime scene

Turan, _________________.

There is a very ruthless killer who kills people without blinking an eye, standing beside a murderer, climbing down the ladder while running away from the crime scene. Suddenly,

_________________.

**(b). *Low-similarity*:**

有一位心狠手辣杀人不眨眼的杀手，站在一名校对员旁边，正在顺着梯子逃离作案现场。

突然，_________________。

You yiweixinhenshoula sharenbuzhayande shashou, zhan-zaiyiming

Have one.CL ruthlesskillpeoplenoblink eyeDE killer,stand-ZAI one.CL

jiaoduiyuan pangbian, zheng-zai shun-zhetizi taoli zuoanxianchang.

proofreaderbeside, ASPalong-ZHE ladder escape commit.a.crime scene

Turan, _________________.

(There is a very ruthless killer who kills people without blinking an eye, standing beside a proofreader, climbing down the ladder while running away from the crime scene. Suddenly,

_________________.)

Pictures were drawn with black ink on white paper. The introductory pictures in each set included a picture of the target referent (the killer in Figure 1a and b) and pictures of the two competitors which either belonged to the *high-similarity* condition (the murderer in Figure 1a) or the *low-similarity* condition (the proofreader in Figure 1b). The target referent and the competitors had the same gender. In total, 27 sets of experimental items had male entities, and 8 sets had female entities. The action picture depicted a simple action carried out only by the target referent (e.g., falling down from the ladder). Each action picture involved a different action. Unlike Fukumura et al. (2011, 2013), we included only the target referent in the action picture.

The target referent and the competitor were also introduced in the introductory sentence (1a: the *high-similarity* condition; 1b: the *low-similarity* condition). To make the target referent particularly salient, it was introduced with the focusing existential structure *有一个*(*/you3yi2ge4/*, “There was”), multiple adjectives modifying the target referent, and multiple phrases predicated of the target referent. The non-salient competitor was mentioned second without additional information. In addition, to increase the coherence between the introductory context sentence and the to-be-described action picture, an adverb indicating manner or time of the action was included at the end of the introductory sentence (e.g., *突然, /tu1ran2/*, “suddenly”; *然后, /ran2hou4/*, “then”; *一不小心, /yi2bu4xiao3xin1/*, “accidentally”).

We also created 35 filler items, each comprising two introductory pictures (one corresponding to the linguistically salient entity and one corresponding to the linguistically non-salient entity) and an action picture. The filler items involved 21 additional animate entities and 14 additional inanimate entities. The introductory sentences for the filler items had a similar structure to the experimental sentences (e.g., *有一台非常珍贵很有年代感的留声机，在一列火车旁边，正在播放音乐。突然，______。 /You3 yi4tai2 fei1chang2 zhen1gui4 hen3 you3 nian2dai4gan3 de liu2sheng1ji1, zai4 yi2lie4 huo3che1 pang2bian1, zheng4zai4 bo1fang4 yin1yue4. Tu1ran2, ______./*, “There was a very precious and very classic gramophone, placed beside a train, playing music. Suddenly, ______”).

#### Design

We manipulated similarity (high- vs. low-similarity) between the target referent and the competitor in a within-subjects and within-items design. We created two lists of stimuli using a Latin Square design in a fixed quasi-random order so that participants saw one version of each item: list A had 17 experimental items from the high-similarity condition and 18 from the low-similarity condition, and list B had the opposite distribution. Both lists contained the 35 filler items.

#### Procedure

Participants were randomly assigned to one of the lists. For those participants assigned to one list, one half saw stimuli in one order and the other half in the reverse order. Participants were tested individually in a laboratory booth. They were given instructions followed by three practice trials. Each trial started with a fixation dot (for 300 ms), followed by the introductory pictures with the target referent on the left and the competitor on the right (again, to emphasise the target referent). The introductory sentence was shown below the introductory pictures, all in the top half of the screen. Participants were asked to view the introductory pictures and read the introductory sentence aloud immediately when they saw it. They then pressed a key to trigger the action picture, which appeared in the bottom half of the screen. They then read the short adverb below the action picture and produced a continuation of the story by describing the action picture. They were asked to describe the action picture as naturally and quickly as they could after seeing it (but there was no time limit). The experiment was run using OpenSesame 1. Responses were digitally recorded. The experiment took around 25–30 min.

#### Scoring

A response was scored as an omission if no explicit subject was mentioned and the sentence started with the main verb; as a pronoun if *他/ 她, /ta1/*, “He”/“She” was mentioned as the subject; or as a repeated NP if the original target referent’s name was mentioned as the subject, regardless of whether any modifiers were mentioned. We excluded all the responses when (a) the subject was the competitor (e.g., *校对员, /jiao4dui4yuan2/*, “proofreader,” *n* = 3); (b) the subject was any other object in the action picture (e.g., *梯子, /ti1zi/*, “the ladder,” *n* = 179); (c) the subject was any other NP, even if it might have referred to the target referent (e.g., *男人, /nan2ren2/*, “that man,” *n* = 14); (d) the participant failed to respond or described an irrelevant event (*n* = 14). In total, we excluded 210 responses (17%).

#### Analyses

Because the dependent variable was categorical (omission vs. non-omission, pronoun vs. non-pronoun, repeated NP vs. non-repeated NP), statistical analysis was carried out using logit mixed-effects models in the lme4 package (version 1.1.20; Bates et al., 2015) in the statistical software R (version 3.5.2) for each referential expression type (the glmer function). For the omission analysis, the outcome variable was omission responses versus non-omission responses; for the pronoun analysis, the outcome variable was pronoun responses versus non-pronoun responses; for the repeated NP analysis, the outcome variable was repeated NP responses versus non-repeated NP responses.

We included Similarity (reference level: low- vs. high-similarity) as the fixed predictor, which were treatment coded (−0.5, 0.5) and centred so that we could interpret the coefficients in a similar way to the main effects and interactions in ANOVAs (Baayen, 2008). The manipulated variables were treated as a within-subjects and within-items factor. The manipulated variable was treated as a within-subjects and within-items factor. We always started with the maximal random structure (Barr et al., 2013), which contained by-subject and by-item random intercepts and slopes for Similarity, plus the correlations between the random slopes and intercepts. When the maximal model failed convergence or resulted in singular fit, we first removed the correlations among the random effects, starting from those of ±1 (Singmann & Kellen, 2019). In the case of the by-subject and by-item correlations were both ±1, we first removed items and then subjects. If the model was still non-converging or had a singular fit, we continued simplifying the random effect based on the covariance–variance matrices of the maximal model, starting with the random effect with the lowest estimated variance. If that was again non-converging, we removed the other one with the second-lowest estimated variance, until the model converged (Singmann & Kellen, 2019). We always made sure that the removal was justified by a likelihood ratio test, where the model with the random effect of interest was compared against the same model without that random effect (Baayen et al., 2008). For all fixed-effects analyses, *p* values were obtained using log-likelihood ratio chi-square tests which compared the model with the fixed effect Similarity with the one without it.

### Results

**Figure 2.**
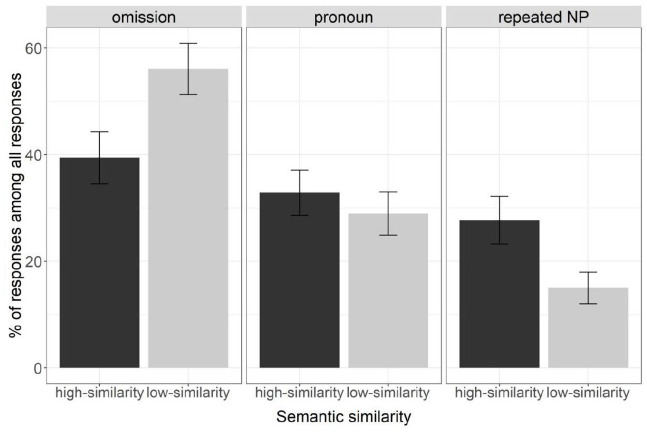
Mean percentages of each referential expression type (omission, pronoun, and repeated NP) out of all responses in Experiment 1. Bars represent standard error of means.

Figure 2 presents the mean percentage of each referential expression type (omission, pronoun, and repeated NP) out of all responses by condition (high- vs. low-similarity) in Experiment 1. Table 1 reports a summary of the coefficients from the analyses on each referential expression type.

**Table 1.** Summary of the logit mixed-effects models results (coefficients β, standard errors *SE*, *z*-values, and *p*-values) on each referential expression type in Experiment 1.

Response types	Predictor	*B*	*SE*	*z*	*p*
Omission^a^	(Intercept)	–0.20	0.34	–0.58	.56
	Semantic similarity	–0.97	0.21	–4.60	<.001
Pronoun^b^	(Intercept)	–1.14	0.29	–3.95	<.001
	Semantic similarity	0.16	0.18	0.85	.397
Repeated NP^c^	(Intercept)	–2.21	0.38	–5.87	<.001
	Semantic similarity	1.10	0.20	5.53	<.001

*SE*: standard error.

The best fit model for ^a^Model and ^b^Model contained by-subject and by-item intercepts, and a by-item slope for Semantic similarity as random effects, ^c^Model contained by-subject and by-item intercepts as random effects.

Participants produced significantly fewer omissions in the high-similarity condition (39%, *n* = 186) than in the low-similarity condition (56%, *n* = 280), χ^2^(1) = 17.08, *p* < .001. They did not differ reliably in their production of pronouns between the high-similarity condition (33%, *n* = 170) and the low-similarity condition (29%, *n* = 157), χ^2^(1) = 0.69, *p* = .406. They produced significantly more repeated NPs (28%, *n* = 143) in the high-similarity condition than in the low-similarity condition (15%, *n* = 79), χ^2^(1) = 30.62, *p* < .001.

### Discussion

Experiment 1 showed that referential behaviour in Mandarin was influenced by the semantic similarity between the target referent and a competitor in the discourse. When the context contained both the target referent and the competitor but the target description involved only the target referent, Mandarin speakers produced more explicit referential expressions (repeated NPs) more frequently, and omitted arguments less frequently, when the competitor was semantically more similar to the target referent than when it was semantically less similar. The finding that semantic similarity led speakers to be more explicit is consistent with the conclusion of Fukumura et al. (2011, 2013) but not with the conclusion of Gennari et al. (2012) and Hsiao et al. (2014). However, the way in which speakers manifested greater explicitness differed from Fukumura et al.’s studies. In our experiment, similarity affected whether speakers produced repeated NPs or omissions, but did not affect whether they produced pronouns; whereas in Fukumura et al.’s studies, similarity affected whether speakers produced repeated NPs or pronouns. We consider this issue in section “General discussion”.

## Experiment 2

In Experiment 2, we used the same target and competitor referents, but with an experimental structure that was different (and so involved different production conditions) from Experiment 1. Specifically, participants first viewed a picture that introduced only the target referent and read aloud a sentence mentioning that entity. They then described a picture in which the target entity performed an action on the competitor, which was a semantically similar or dissimilar entity. This experimental structure is comparable with Hsiao et al. (2014) and Gennari et al. (2012), with respect to the involvement of both the target and competitor in the target event.

If the effects of semantic similarity depend on production conditions relating to variations in the retrieval difficulty of the entities, then we would expect to find a different pattern of effects than Experiment 1, given that in Experiment 2 only the target referent was introduced but both entities needed to be mentioned; specifically, we would expect the pattern consistent with Hsiao et al. (2014) and Gennari et al. (2012). Thus, we predicted that under these conditions, speakers would produce more referring expressions that are less explicit and fewer referring expressions that are more explicit to refer to the target referent when the target referent and competitor were semantically similar than when they were dissimilar—the opposite pattern from Experiment 1. If this pattern of results is found, we will discuss the choice between pronouns and repeated NPs in the General Discussion.

### Method

#### Participants

Thirty-five further participants from the same population as Experiment 1 took part (3 males; *M_age_* = 23 years).

#### Items

**2. High-similarity and low-similarity:**

有一位心狠手辣杀人不眨眼的杀手，正在顺着梯子逃离作案现场。突然，

_________________。

Youyiweixinhenshoulasharenbuzha yande shashou, zheng-zai shun-zhe

Have one.CL ruthlesskillpeoplenoblink eyeDE killer, ASP along-ZHE

tizi taoli zuoan xianchang.

ladder escape commit.a.crime scene

Turan, _________________.

(There is a very ruthless killer who kills people without blinking the eye, climbing down the ladder while running away from the crime scene. Suddenly, _________________.)

**Figure 3.**
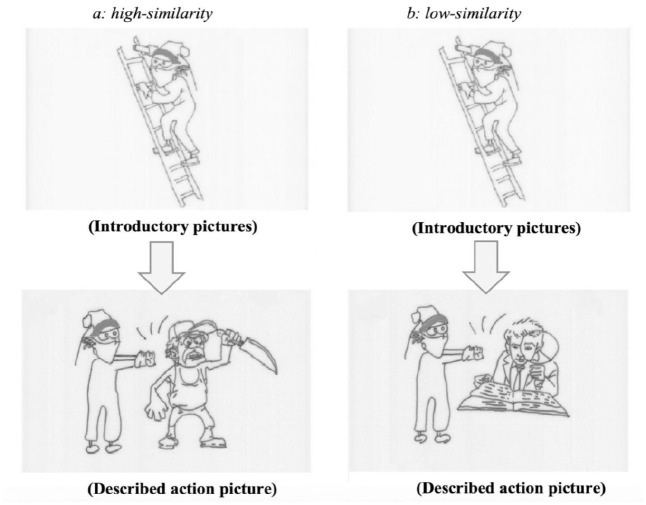
Example experimental pictures in the similarity conditions (Experiment 2): (a) high-similarity and (b) low-similarity.

The same 70 target referent-competitor pairs as in Experiment 1 were used, leading to 35 sets of experimental items in total. Differently from Experiment 1, each set included only one introductory picture (top panels in Figure 3a and b) and one introductory sentence (2) mentioning only the target referent (*the killer*). The introductory sentences were constructed by removing the second clause mentioning the competitor from the introductory sentences used in Experiment 1 (e.g., *standing beside a murderer/proofreader* in 1). Each set also contained two action pictures (bottom panels in Figure 3a and b), depicting a simple action carried out by the target referent on the competitor such as kicking, pushing, hitting (*high similarity* condition: the killer pushed the murderer; *low-similarity* condition: the killer pushed the proofreader). In total, we used 10 action verbs. Each picture was drawn with black ink on white paper.

We used the same 35 filler pictures as in Experiment 1. But unlike Experiment 1, each set of filler items comprised only one introductory picture and one action picture so that their presentation was consistent with that of experimental items. The introductory sentences for the filler items had the same structure as the experimental items (e.g., *有一台非常珍贵很有年代感的留声机，正在播放音乐。突然，______。 /You3 yi4tai2 fei1chang2 zhen1gui4 hen3 you3nian2dai4gan3 de liu2sheng1ji1, zheng4zai4 bo1fang4 yin1yue4. Tu1ran2*, ______.*/*, “There was a very precious and very classic gramophone, playing music”).

#### Design

The design was identical to Experiment 1.

#### Procedure

Participants were tested individually in a laboratory booth. A familiarisation session was added prior to the main experiment, during which participants were familiarised with the competitor pictures that would appear in the target events in the experiment (as they would not be presented in the introductory picture or the introductory sentence). They were first presented with the 35 competitor pictures in sequence, with corresponding occupation names labelled below each picture. Participants pressed a key to view the next picture. They were instructed to study the pictures and the corresponding occupation names at their own pace. Each picture was shown twice. Participants’ memory was then tested: They viewed the pictures in a different order and named all the pictures without the labels. The experimenter corrected participants if they used a different name from the label.

After the familiarisation session, participants proceeded immediately to the main experiment. Unlike Experiment 1, on each trial, after a fixation dot (300 ms), participants viewed a single introductory picture depicting the target referent and read aloud the introductory sentence mentioning the same target referent, which appeared on the top half of the screen. The action picture was subsequently trigged and described in the same way as in Experiment 1.

#### Sentence scoring and analyses

These were identical to Experiment 1. In total, we excluded 282 responses (27%). Like Experiment 1, we excluded responses when (a) the subject was the competitor (e.g., *校对员, /jiao4dui4yuan2/*, “proofreader,” *n* = 176); (b) the subject was any other object in the action picture (e.g., *梯子, /ti1zi/*, “the ladder,” *n* = 60); (c) the subject was any other NP, even if it might have referred to the target referent (e.g., *男人, /nan2ren2/*, “that man,” *n* = 46); (d) the participant failed to respond (*n* = 0).

### Results

**Figure 4.**
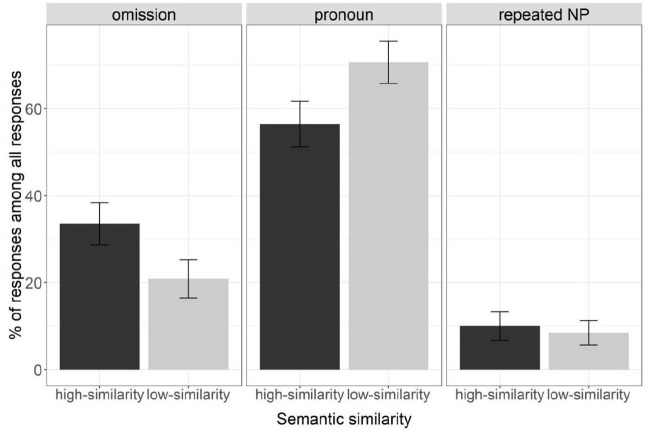
Mean percentages of each referential expression type (omission, pronoun, and repeated NP) out of all responses in Experiment 2. Bars represent standard error of means.

Figure 4 presents the mean percentage of responses of each type (omission, pronoun, and repeated NP) out of all responses by condition (high- and low-similarity) in Experiment 2. Table 2 reports a summary of the coefficients from the analyses on each response type.

**Table 2.** Summary of the logit mixed-effects models results (coefficients β, standard errors *SE*, *z*-values, and *p*-values) on each referential expression type in Experiment 2.

Response types	Predictor	β	*SE*	*z*	*p*
Omission^a^	(Intercept)	–1.63	0.37	–4.46	<.001
	Semantic similarity	0.96	0.19	5.11	<.001
Pronoun^b^	(Intercept)	0.84	0.35	2.39	.017
	Semantic similarity	–0.98	0.18	–5.49	<.001
Repeated NP^c^	(Intercept)	–4.29	0.64	–6.66	<.001
	Semantic similarity	0.29	0.32	0.91	.361

*SE*: standard error.

The best-fitted model for ^a^Model, ^b^Model and ^c^Model all contained by-subject and by-item intercept as random effects.

Participants produced more omissions in the high-similarity condition (34%, *n* = 163) than the low-similarity condition (21%, *n* = 108), χ^2^(1) = 26.09, *p* < .001. They produced fewer pronouns in the high-similarity condition (56%, *n* = 259) than in the low-similarity condition (71%, *n* = 327), χ^2^(1) = 30.51, *p* < .001. Their production of repeated NPs did not differ reliably between the high-similarity condition (10%, *n* = 46) and the low-similarity condition (8%, *n* = 10), χ ^2^(1) = 0.84, *p* = .361.

### Discussion

Experiment 2 again showed that referential behaviour in Mandarin was influenced by the semantic similarity between the target referent and a competitor in the discourse. However, the pattern of effects differed from Experiment 1. When the context contained only the target referent, but the target event involved both the target referent and the competitor, Mandarin speakers were less referentially explicit: They omitted the target referent more frequently, and used pronouns less frequently, when the competitor was semantically more similar to the target referent than when it was semantically less similar. The finding that semantic similarity led speakers to be less explicit is consistent with Gennari et al. (2012) and Hsiao et al. (2014), but not with Fukumura et al. (2011, 2013).

## The effects of phonological similarity in Experiments 1 and 2

Experiments 1 and 2 manipulated semantic similarity, and showed effects of this manipulation on speakers’ choice of referential expressions. Moreover, we have argued that such semantic similarity effects might occur at different stages of processing, namely during discourse processing during the selection of entities and lemma processing during the selection of the lexical items. However, lexical processing also involves choice of word form, during which phonological information is retrieved (Levelt et al., 1999). Might competition between phonologically similar entities occur at this stage? Researchers have found that phonological similarity can affect lexical access in picture-word interference studies (e.g., Zhang et al., 2009) and it can sometimes affect syntactic choice during sentence production (Bock, 1987; though cf. Bock, 1986). If this is the case, phonological overlap between the target-competitor pairs (with respect to their component syllables, in the same or different position within the words) should also yield interference, and this interference might also affect speakers’ choice of referring expressions.

We did not design stimuli to investigate phonological effects, and so we conducted an exploratory analysis of whether phonological similarity affected speakers’ choice of referential expression in Experiments 1 and 2. It is also possible that phonological similarity might explain some of the effects that we have attributed to semantic similarity, and we therefore included semantic as well as phonological similarity in this analysis. Like semantic similarity, we coded phonological similarity as a categorical variable. Target–competitor pairs were coded as phonologically similar if they contained at least one overlapping syllable (i.e., both the phonemes and the tone were the same), irrespective of whether the overlapping syllable(s) occurred in the same position within the word (e.g., *海盗, /hai3dao4/*, “pirate” & *强盗, /qiang1dao4/*, “burglar” where the sound overlaps in the second syllable) or in different positions within the word (e.g.,*画家, /hua4jia1/*, “painter” & *插画师, /cha1hua4shi1/*, “illustrator” where the sound overlaps in the first syllable in the former but the second syllable in the latter). Otherwise, they were coded as phonologically dissimilar. In total, 23 pairs were coded as phonologically similar (22 were semantically similar and one was semantically dissimilar), and 47 were coded as phonologically dissimilar (13 were semantically similar and 34 were semantically dissimilar).

### Experiment 1

#### Analyses

Because the outcome variables were categorical (omission vs. non-omission, pronoun vs. non-pronoun, repeated NP vs. non-repeated NP), statistical analysis was carried out using logit mixed-effects models in the lme4 package (version 1.1.20; Bates et al., 2015) in the statistical software R (version 3.5.2) for each referential expression type (the glmer function). However, there were no repeated NPs when the target and competitor were phonologically similar but semantically dissimilar (e.g., 杂技演员, /za2ji4yan3yuan2/, “acrobat” vs. 图书管理员, /tu2shu1guan3li3yuan2/, “librarian”). This situation causes the maximum likelihood estimate (Wald’s Test) of a logistic mixed effects (LMEs) regression models to tend towards infinity for the outcome variable (Hauck & Donner, 1977). We therefore used Bayesian logistic mixed effects (BLMEs) models when analysing repeated NPs, as it can improve parameter estimates for repeated NP responses.

The models included Phonological similarity (similar vs. dissimilar) and Semantic similarity (similar vs. dissimilar) as fixed effects, which were centred prior to analysis (Baayen, 2008), and subjects and items as random effects (Baayen et al., 2008). Details of model selection followed the same approach as in main analyses of Experiments 1 and 2.

#### Results and discussion

Table 3 presents the mean percentage of each referential expression type (omission, pronoun, and repeated NP) out of all responses by Phonological similarity (similar vs. dissimilar) in Experiment 1. Table 4 reports a summary of the coefficients from the analyses for each referring expression type.

**Table 3.** Percentages and standard deviations (*SD*) of each referential expression type (omission, pronoun, and repeated NP) by Phonological similarity out of all responses in Experiment 1.

Phonological similarity	Referential expression type	Percentage (%)	*SD*
Similar	Omission	40.30	0.49
	Pronoun	31.60	0.47
	Repeated NP	28.10	0.45
Dissimilar	Omission	48.40	0.50
	Pronoun	32.50	0.47
	Repeated NP	19.10	0.39

*SD*: standard deviation; NP: noun phrase.

**Table 4.** Summary of the logit mixed-effects models results (coefficients β, standard errors *SE*, *z*-values, and *p*-values) for each referential expression type in Experiment 1.

Response types	Predictor	β	*SE*	*z*	*p*
Omission^a^	(Intercept)	0.45	0.46	0.97	.33
	Phonological similarity	2.44	1.15	2.12	.034
	Semantic similarity	–2.43	0.74	–3.27	<. 01
	Phonological similarity: semantic similarity	–4.35	2.18	–1.99	<.05
Pronoun^b^	(Intercept)	–1.43	0.37	–3.88	<.001
	Phonological similarity	–1.33	0.82	–1.62	.105
	Semantic similarity	0.93	0.52	1.78	.075
	Phonological similarity: semantic similarity	1.88	1.53	1.23	.220
Repeated NP^c^	(Intercept)	–2.43	0.45	–5.3	<.001
	Phonological similarity	–0.58	0.86	–0.68	.500
	Semantic similarity	1.43	0.54	2.61	<.01
	Phonological similarity: semantic similarity	1.37	1.59	0.86	.391

*SE*: standard error.

The best fit model for ^a^Model and ^b^Model contained by-subject and by-item intercepts as random effects, ^c^Model contained by-subject and by-item intercept, and a by-item slope for Phonological similarity as random effects.

**Figure 5.**
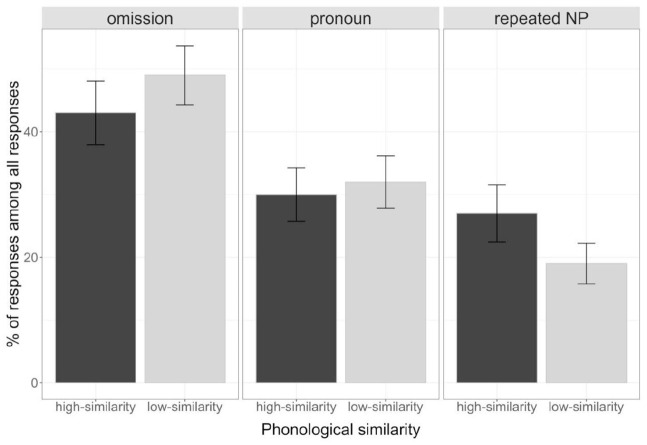
Mean percentages of each referential expression type (omission, pronoun, and repeated NP) by Phonological similarity out of all responses in Experiment 1. Bars represent standard error of means.

As Table 4 shows, these exploratory analyses revealed no main effects of phonological similarity in the production of pronouns or repeated NPs. They did reveal a main effect of phonological similarity for omissions, with participants producing more omissions for the phonologically dissimilar than similar referential expressions, and an interaction between phonological and semantic similarity. Additional analyses indicated that they produced significantly fewer omissions in the phonologically and semantically similar condition than the other conditions, χ^2^(1) = 0.19, *p* = .046. But more importantly, the analyses revealed the same effects of semantic similarity as in the analyses above: more omissions in the semantically dissimilar than similar condition, and more repeated NPs in the semantically similar than dissimilar condition. In sum, these additional analyses are consistent with our conclusions about the effects of semantic similarity on choice of referring expression (Figure 5).

### Experiment 2

#### Analyses

These were identical to the corresponding analyses for Experiment 1 reported in section “Analyses” (except that we used LME4 for repeated NPs because there was no missing response category of repeated NPs).

#### Results and discussion

Table 5 presents the mean percentage of each referential expression type (omission, pronoun, and repeated NP) out of all responses by Phonological similarity (similar vs. dissimilar) in Experiment 2. Table 6 reports a summary of the coefficients from the analyses on each referring expression type.

**Table 5.** Percentages and standard deviations (*SD*) of each referential expression type (omission, pronoun, and repeated NP) by Phonological similarity out of all responses in Experiment 2.

Phonological similarity	Referential expression type	Percentage (%)	*SD*
Similar	Omission	35.90	0.48
	Pronoun	56.80	0.50
	Repeated NP	7.30	0.26
Dissimilar	Omission	25.20	0.43
	Pronoun	64.80	0.48
	Repeated NP	10.00	0.30

*SD*: standard deviation; NP: noun phrase.

**Table 6.** Summary of the logit mixed-effects models results (coefficients β, standard errors *SE*, z-values, and *p*-values) on each referential expression type in Experiment 2.

Response types	Predictor	β	*SE*	*Z*	*p*
Omission^a^	(Intercept)	–1.86	0.41	–4.54	<.001
	Phonological similarity	–0.33	0.59	–0.56	.574
	Semantic similarity	1.16	0.42	2.75	<.01
	Phonological similarity: semantic similarity	1.50	1.17	1.28	.199
Pronoun^b^	(Intercept)	*1.00*	*0.40*	*2.48*	<.05
	Phonological similarity	0.47	0.60	0.78	.433
	Semantic similarity	–1.26	0.41	–3.10	<.01
	Phonological similarity: semantic similarity	–0.97	1.18	–0.82	.413
Repeated NP^c^	(Intercept)	–4.05	0.67	–6.03	<.001
	Phonological similarity	–0.06	0.82	–0.07	.946
	Semantic similarity	0.27	0.57	0.47	.636
	Phonological similarity: semantic similarity	–1.57	1.55	–1.01	.313

*SE*: standard error.

The best fit model for ^a^Model and ^b^Model contained by-subject and by-item intercepts, and a by-item slope for Phonological similarity as random effects. ^c^Model contained by-subject and by-item intercepts, and a by-item slope for Phonological similarity as random effects.

**Figure 6.**
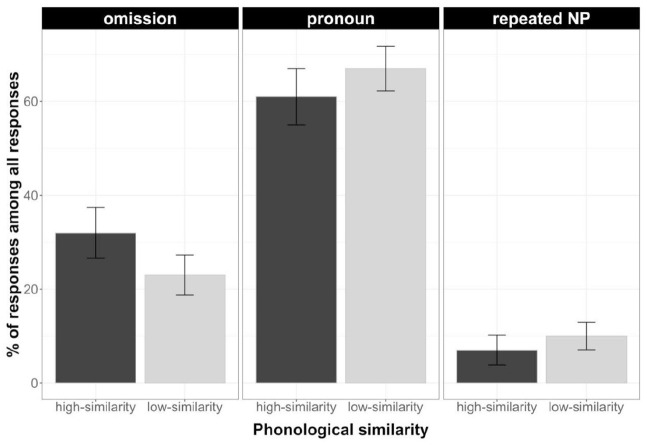
Mean percentages of each referential expression type (omission, pronoun, and repeated NP) by Phonological similarity out of all responses in Experiment 2. Bars represent standard error of means.

As Table 6 shows, these exploratory analyses revealed no effects of phonological similarity or interactions between phonological and semantic similarity in the production of omissions, pronouns, or repeated NPs. Importantly, they did reveal the same effects of semantic similarity as in the analyses above: more omissions in the semantically similar than dissimilar condition, and more pronouns in the semantically dissimilar than similar condition. In sum, these additional analyses are again consistent our conclusions about the effects of semantic similarity on choice of referring expression (Figure 6).

## General discussion

In two experiments, native Mandarin speakers performed a picture description task involving a target referent and a competitor that were semantically similar or dissimilar. Both experiments showed that similarity affected the choice of referential expressions, but they showed starkly contrasting effects. Experiment 1 found that participants tended to use more explicit referential expressions (repeated NPs) and fewer omissions when the referent and competitor were similar than when they were dissimilar. This pattern is in line with Fukumura and colleagues’ findings (Fukumura et al., 2011, 2013). In contrast, Experiment 2 found that Mandarin speakers tended to use less explicit referential expressions (omissions) and more pronouns when the referent and competitor were similar than when they were dissimilar. This pattern is in line with Gennari et al. (2012) and Hsiao et al. (2014).

Our results suggest that the contrasting effects of semantic similarity were not due to the difference in the choice of referential expressions between the two sets of results (i.e., relating to the fact that omission occurred in Hsiao et al., 2014, but not in Fukumura et al., 2011). If this had been the case, our experiments should have produced results similar to Hsiao et al. (2014).

Instead, we propose the contrasting effects of semantic similarity might arise from another difference between the two studies: whether both the target and competitor entities needed to be described in the target event (Hsiao et al., 2014) or only the target entity was relevant (Fukumura et al., 2011). Of course, there are other differences between the experiments, which reflect our goal of reproducing the key characteristics of the two sets of studies that had yielded apparently conflicting results. For example, the distance between the target referent in the context and target sentences is greater in Experiment 1 than Experiment 2 (in keeping with the characteristics of Fukumura et al.’s and Hsiao et al.’s studies, respectively). These differences might lead to differences in the overall distribution of referring expressions between the two experiments, but do not provide a clear explanation for the distribution. In contrast, we suggest that they can be explained in terms of the process of language production, as we now discuss.

### Different stages of the effects of semantic similarity

Why might we find these contrasting effects of semantic similarity associated with the number of entities that needed to be described in the target event? We argue that those different conditions cast light on speakers’ different ways of processing the discourse entities, so that the effects of semantic similarity occurred at different stages of production: discourse processing (Experiment 1) versus linguistic processing (Experiment 2).

In Experiment 1, the context contained both the target referent and a semantically similar or dissimilar competitor, but the target event involved only the target referent. We propose that under such conditions, the effects of semantic similarity started as early as the initial discourse processing level where the entities’ non-linguistic conceptual representations are encoded, because semantic similarity generated interference by reducing the conceptual accessibility of the target referent, which in turn affected speakers’ linguistic choice of referential expressions. That is, when participants read the context, they established a discourse model containing two entities. The structure of the context sentence (e.g., first mention, predication) made the target entity highly activated. Furthermore, the activation of the target entity was lower when the competitor was semantically similar to it than when it was dissimilar to it. We assume this was because in the high-similarity condition, activation from semantic feature nodes that were associated with the target referent (e.g., +KILL) was split between the target and competitor entities (because the competitor entities are also associated with that feature node). Activation helps speakers access the referent (Griffin, 2010), and so the target referent was less accessible in the high- than the low-similarity condition.

Participants then had to produce the critical sentence by referring to the target entity. To do this, they needed to consult their discourse model because the choice of referential expression is determined by the activation of the target entity within the discourse model following the accessibility hierarchy (e.g., Ariel, 1990; Chafe, 1976)—speakers tend to refer to less highly activated entities with more explicit referential expressions. For example, they are more likely to use a full NP to refer to a distant antecedent (Ariel, 1990). In the same way, they are more likely to use a full NP when referring to an antecedent that is semantically similar to its alternative.

In the low-similarity condition of Experiment 1, the target entity’s representation was strongly activated (because no retrieval interference would be generated due to the dissimilar competitor) and so participants did not consider a full NP because it would not be felicitous to use such an expression. Instead, they used the less explicit omissions. In the high-similarity condition, in contrast, the target entity’s representation was not strongly activated and so the participant often chose a full NP as an appropriate form of referential expression. Therefore, in Experiment 1, participants tended to use more repeated NPs (and fewer omissions) in the high-similarity condition than in the low-similarity condition.

Participants then linguistically encoded the target entity, during which its lemma was first retrieved (Levelt, 1989). One might argue that the effects of semantic similarity might spread into this later level. However, we speculate that in Experiment 1, there would in practice be little such interference during lemma processing, because the target entity was the only entity involved in the target event in each trial and this lemma processing did not implicate the competitor entity. At this stage it was therefore not relevant whether the competitor was semantically similar to the target referent. In other words, semantic similarity effects were unlikely to occur during lemma processing in Experiment 1.

To sum up, participants in Experiment 1 showed a tendency to produce more explicit (rather than less explicit) referring expressions when the competitor was semantically similar to the target referent compared with when it was semantically dissimilar, a pattern consistent with Fukumura and colleagues’ findings (Fukumura et al., 2011, 2013). However, given that both omissions and pronouns are comparatively implicit referring expressions (i.e., the name of the target referent is not ultimately articulated), why does semantic similarity affect the production of omissions, but not pronouns? One possibility is the overwhelmingly high use of pronouns in spoken Mandarin, compared with omissions and repeated NPs (M. B. Christensen, 2000), which may give the speaker a “more personal and conversational flavor” (p. 328). Such a property might override any effects of semantic similarity on the use of pronouns, resulting in no effect on pronouns in Experiment 1.

In Experiment 2, in contrast, the context contained only the target referent, but the target event involved both the target referent and the competitor. We propose that under such production conditions, semantic similarity was unlikely to affect discourse processing, because there was only one entity in the discourse (the target referent). So, when participants processed the context, they established a discourse model containing only a single target entity. Therefore, the activation of the target entity remained very high in both the high- and low-similarity conditions (unlike in Experiment 1).

The participants then had to generate the critical sentence by referring to the target entity and the competitor entity. To do this, they first consulted their discourse model. Because the target entity was always highly accessible, they did not consider a full NP as a possible referential expression in either high- or low-similarity conditions, because this would not be a felicitous form (following the accessibility hierarchy; Ariel, 1990). Thus, semantic similarity was therefore not relevant to discourse processing in Experiment 2.

Instead, we argue that the effects of semantic similarity occurred at the subsequent lemma processing level because the speaker has the (new) task of linguistically encoding the competitor. Crucially, this process is more difficult when the competitor is similar to the target referent than when it is dissimilar to the target referent (because of semantic interference, as occurs in picture–word interference studies; Lupker, 1979; Schriefers et al., 1990). In these circumstances, the speaker should refer to the competitor entity using a full NP (because it is not already present in the discourse representation, hence inaccessible under the accessibility hierarchy) and so must select and retrieve its associated lexical entry. In the high-similarity condition, this lexical entry competes with the target entity’s lexical entry and therefore inhibits its retrieval, in comparison with the low-similarity condition. But because the target is compatible with an omission or a pronoun, processing difficulty is minimised by using the least explicit referential expression (an omission) to refer to the target referent, as this does not require retrieval of the wordform (and perhaps lemma)—the pattern that we found in Experiment 2.

Note that our results are not due to ambiguity avoidance. The target and the competitor always had the same gender in the current study. Thus, pronouns, as well as omissions, are globally ambiguous regardless of the semantic similarity between the target referent and the competitor, and they could be interpreted as referring to either of the entities. Therefore, if the effect of semantic similarity had been driven by ambiguity avoidance, the use of pronouns and omissions should not have differed between the two similarity conditions. However, our results disconfirmed this prediction as we observed a difference in the use of omissions (Experiment 1) and pronouns (Experiment 2) between the high-similarity and low-similarity condition. Instead, our results are more consistent with the semantic competition account. It suggests that speakers were affected by semantic similarity because a high-similarity competitor interferes more strongly with the target referent than a low-similarity competitor.

### Phonological similarity

Our additional analyses suggest that phonological similarity cannot explain our results. When we included a measure of phonological similarity, we again found the same patterns of semantic similarity effects as in the main analyses. Moreover, we found no effect of phonological similarity for pronouns or repeated NPs in Experiment 1, and no effect of phonological similarity for any response type in Experiment 2. In Experiment 1, there was a main effect of phonological similarity and an interaction between phonological similarity and semantic similarity on omissions—but crucially, there was still a main effect of semantic similarity when phonological similarity was controlled. Hence we can be confident that our conclusions regarding effects of semantic similarity still hold. The additional analyses suggest that under some circumstances, phonological similarity might also play a role in choice of referential expressions. This suggests an interesting direction for future research. Our study was not designed to test phonological similarity effects, but future studies could manipulate phonological similarity systematically to investigate this.

To conclude, our study showed that Mandarin speakers experience interference caused by the semantic similarity between the target referent and the competitor in referential production. Crucially, we found the consequences of the effects of semantic similarity is dependent on production conditions. When only the target had to be mentioned, Mandarin speakers preferred to use more explicit referential expressions (repeated NPs). In contrast, when both the target and the competitor had to be mentioned, Mandarin speakers preferred to use less explicit referential expressions (omissions). We argue that the contrasting findings occur because semantic similarity affects different stages of production: discourse processing versus linguistic processing.

## Supplemental Material

sj-docx-1-qjp-10.1177_17470218231154578 – Supplemental material for The effects of semantic similarity on Mandarin speakers’ referential expressions Click here for additional data file. Supplemental material, sj-docx-1-qjp-10.1177_17470218231154578 for The effects of semantic similarity on Mandarin speakers’ referential expressions by Yangzi Zhou, Holly P Branigan, Yue Yu and Martin J Pickering in Quarterly Journal of Experimental Psychology
