# Supplementary material for: The effects of semantic similarity on Mandarin speakers’ referential expressions
Source: Q J Exp Psychol (Hove). 2023 Mar 1;76(11):2579–95. doi: 10.1177/17470218231154578 (PMC10585944; doi:10.1177/17470218231154578)
Supplement: sj-docx-1-qjp-10.1177_17470218231154578 – Supplemental material for The effects of semantic similarity on Mandarin speakers’ referential expressions [file sj-docx-1-qjp-10.1177_17470218231154578.docx]

Supplementary Material for:

**The effects of semantic similarity on Mandarin speakers’ referential expressions**

Yangzi Zhou

Holly P. Branigan

Yue Yu

Martin J. Pickering

*Department of Psychology*

*University of Edinburgh*

Corresponding author:

Yangzi Zhou (yangzi.zhou88@yahoo.com)

7 George Square, Edinburgh, EH8 9AD, United Kingdom

**Supplementary Material: Context sentences for Experiments 1 and 2**

Each experimental item included a context sentence (in lower case), followed by a target event depicted in the action pictures (in italics). (a) represents the stimuli used in Experiment 1, and (b) represents the stimuli used in Experiment 2. Chinese texts are in square brackets. The targets are in bold and the competitors are underlined. Those competitors before the slash occurred in the high-similarity condition and those after the slash occurred in the low-similarity condition. In Experiment 1, context sentences mentioned both the targets and the competitors, and the target events involved only the targets. In Experiment 2, context sentences mentioned only the targets, and the target events involved both the targets and the competitors. The mean similarity ratings for each condition are shown in brackets after each competitor in (a).

1a. There is a very fierce **pirate** with a big knife, standing next to a robber (5.5)/cashier (1.6), giving orders on board. ***Pirate*** *falling off the ship.*

[有一位看上去特别凶手里举着把大刀的**海盗**，站在一名强盗/收银员旁边，正在船上发号施令。***海盗****从船上掉了下来。*]

1b. There is a very fierce **pirate** with a big knife, giving orders on board. ***Pirate*** *pulling robber/cashier.*

[有一位看上去特别凶手里举着把大刀的**海盗**，正在船上发号施令。***海盗****拉了一下强盗/收银员。*]

2a. There is a **prisoner** in fetters and a prison uniform, sitting next to a criminal (5.6)/decorator(2.0), sleeping. ***Prisoner*** *falling off the bed.*

[有一位戴着脚镣穿着囚服的**囚犯**，坐在一名罪犯/装潢师旁边，正在睡觉。***囚犯****从床上掉了下来。*]

2b. There is a **prisoner** with fetters and a prison uniform, sleeping. ***Prisoner*** *pinching criminal/decorator.*

[有一位戴着脚镣穿着囚服的**囚犯**，正在睡觉。***囚犯****掐了一下罪犯/装潢师。*]

3a. There is a very young and hardworking **farmer**, standing next to a shepherd (4.9)/sculptor (2.2), driving a wagon. ***Farmer*** *falling off the wagon.*

[有一位很年轻很勤劳的**农场主**，站在一名牧羊人/雕刻家旁边，正在赶马车。**农场主**从马车上摔了下来。]

3b. There is a very young and hardworking **farmer**, driving a wagon. ***Farmer*** *kicking shepherd/sculptor.*

[有一位很年轻很勤劳的**农场主**，正在赶马车。***农场主****踢了一下牧羊人/雕刻家。*]

4a. There is a very insidious and bad **blackmailer**, standing next to a swindler (5.3)/typist (1.9), blackmailing passengers on the train. ***Blackmailer*** *jumping off the train.*

[有一位非常阴险非常坏的**敲诈犯**，站在一名诈骗犯/打字员旁边，正在敲诈火车上的乘客。***敲诈犯****从火车上跳了下来。*]

4b. There is a very insidious and bad **blackmailer**, blackmailing the passengers on the train. ***Blackmailer*** *hitting swindler/typist.*

[有一位非常阴险非常坏的**敲诈犯**，正在敲诈火车上的乘客。***敲诈犯****打了一下诈骗犯/打字员。*]

5a. There is a very vivacious somewhat short **clown**, standing next to a comedian (5.5)/operator(2.5), juggling on the steps. ***Clown*** *falling down the steps.*

[有一位特别活泼个子有些矮的**小丑**，站在一名喜剧演员/接线员旁边，正在台阶上表演杂耍。***小丑****从台阶上摔了下来。*]

5b. There is a very vivacious somewhat short **clown**, juggling on the steps. ***Clown*** *touching comedian/operator.*

[有一位特别活泼个子有些矮的**小丑**，正在台阶上表演杂耍。***小丑****碰了一下喜剧演员/接线员。*]

6a. There is a **thief** who is addicted to theft and has great courage, sitting next to a burglar (6.7)/astronaut (1.6), thinking about the crime plan. ***Thief*** *jumping up from the seat.*

[有一位盗窃成瘾胆子特别大的**小偷**，坐在一名窃贼/宇航员旁边，正在思考作案方案。***小偷****从座位上跳了起来。*]

6b. There is a **thief** who is addicted to theft and has great courage, thinking about the crime plan. ***Thief*** *hitting burglar/astronaut.*

[有一位盗窃成瘾胆子特别大的**小偷**，正在思考作案方案。***小偷****打了一下窃贼/宇航员。*]

7a. There is a ruthless **killer** who kills people without blinking an eye, standing beside a murderer (6.2)/proofreader (1.7), climbing down the ladder while running away from the crime scene. ***Killer*** *falling off the ladder.*

[有一位心狠手辣杀人不眨眼的**杀手**，站在一名杀人犯/校对员旁边，正在顺着梯子逃离作案现场。***杀手****从梯子上掉了下来。*]

7b. There is a ruthless **killer** who kills people without blinking an eye, climbing down the ladder while running away from the crime scene. ***Killer*** *pushing burglar/astronaut.*

[有一位心狠手辣杀人不眨眼的**杀手**，正在顺着梯子逃离作案现场。***杀手****推了一下杀人犯/校对员。*]

8a. There is a very elegant and charming **dancer**, standing next to a choreographer (5.1)/dentist (1.7), practicing the new dance. ***Dancer*** *spraining the ankle.*

[有一位很优雅很有魅力的**舞蹈家**，站在一名编舞者/牙医旁边，正在练习新编的舞蹈。***舞蹈家****崴了脚。*]

8b. There is a very elegant and charming **dancer**, practicing the new dance. ***Dancer*** *pulling choreographer/dentist.*

[有一位很优雅很有魅力的**舞蹈家**，正在练习新编的舞蹈。***舞蹈家****拉了一下编舞者/牙医。*]

9a. There is a very shrewd **businessman** with special temperament, sitting next to an entrepreneur (5.6)/breeder (2.4), looking at the contract. ***Businessman*** *overturning the table.*

[有一位十分精明看上去特别有气质的**商人**，坐在一名企业家/饲养员旁边，正在看合同。***商人****掀翻了桌子。*]

9b. There is a very shrewd **businessman** with special temperament, looking at the contract. ***Businessman*** *kicking entrepreneur/breeder.*

[有一位十分精明看上去特别有气质的**商人**，正在看合同。***商人****踢了一下企业家/饲养员。*]

10a. There is a very young and talented **lecturer** who is very popular with students, standing next to a teacher (5.9)/cowboy (2.3), taking a class. ***Lecturer*** *hitting his head on the blackboard.*

[有一位年轻有才很受学生欢迎的**大学讲师**，站在一名教师/牛仔旁边，正在上课。***大学讲师****头撞到了黑板*]

10b. There is a very young and talented **lecturer** who is very popular with students, taking a class. ***Lecture*** *kicking teacher/cowboy.*

[有一位年轻有才很受学生欢迎的**大学讲师**，正在上课。***大学讲师****踹了一下教师/牛仔。*]

11a. There is a very knowledge **professor** with a lot of books, standing next to a scholar (6.0)/repairman (2.5), preparing a lesson. ***Professor*** *bumping his head against the blackboard.*

[有一位非常有学识拥有很多藏书的**教授**，坐在一名学者/修理工旁边，正在备课。***教授****头撞到了书柜。*]

11b. There is a very knowledge **professor** with a lot of books, preparing a lesson. *Professor pulling scholar/repairman.*

[有一位非常有学识拥有很多藏书的**教授**，正在备课。***教授****拉了一下学者/修理工。*]

12a. There is an **interviewer** who works very seriously and looks a little tired, standing next to an examiner (5.4)/doorman (2.4), reviewing the candidates. ***Interviewer*** *hitting his head on the table.*

[有一位工作特别认真看上去有些疲惫的**面试官**，坐在一名考官/门童旁边，正在审核面试者材料。***面试官****头磕到了桌子。*]

12b. There is an **interviewer** who works very seriously and looks a little tired, reviewing interviewer materials. ***Interviewer*** *stepping on examiner/doorman.*

[有一位工作特别认真看上去有些疲惫的**面试官**，正在审核面试者材料。***面试官****踩了一下考官/门童。*]

13a. There is a very neat and diligent **nanny**, standing next to an hourly worker (5.0)/writer (2.4), preparing to clean the vase. ***Nanny*** *knocking over the vase.*

[有一位非常爱干净干活勤勤恳恳的**保姆**，站在一名钟点工/作家旁边，正在准备擦花瓶。***保姆****碰倒了花瓶。*]

13b. There is a very clean and diligent **nanny**, preparing to clean the vase. ***Nanny*** *kicking hourly worker/writer.*

[有一位非常爱干净干活勤勤恳恳的**保姆**，正在准备擦花瓶。***保姆****踢了一下钟点工/作家。*]

14a. There is a very dedicated and highly skilled **pedicure**, standing next to a manicurist (4.9)/bus driver (1.6), serving guests. ***Pedicure*** *knocking over the sink.*

[有一位非常敬业技艺特别高超的**修脚师**，站在一名美甲师/公交司机旁边，正在为客人服务。***修脚师****碰翻了水池。*]

14b. There is a very dedicated and highly skilled **pedicure**, serving the clients. ***Pedicure*** *pinching manicurist/bus driver.*

[有一位非常敬业技艺特别高超的**修脚师**，正在为客人服务。***修脚师****掐了一下美甲师/公交司机。*]

15a. There is a very athletic and healthy **swimmer**, standing next to a diver (5.9)/vet (2.2), warming up. ***Swimmer*** *diving into the water.*

[有一位特别爱运动非常健康的**游泳者**，站在一名潜水员/兽医旁边，正在做热身。***游泳者****跳入水中。*]

15b. There is a very active and healthy **swimmer**, warming up. ***Swimmer*** *bumping into diver/vet.*

[有一位特别爱运动非常健康的**游泳者**，正在做热身。***游泳者****撞了一下潜水员/兽医。*]

16a. There is a very careful and introverted **knitter**, sitting next to an embroiderer (5.4)/ archaeologist (1.5), knitting a sweater. ***Knitter*** *pricking her hand.*

[有一位十分细心性格有些内向的**针织工**，坐在一名刺绣工/考古学家旁边，正在织毛衣。***针织工****扎到了手。*]

16b. There is a very careful and introverted **knitter**, knitting a sweater. ***Knitter*** *kicking embroiderer/ archaeologist.*

[有一位十分细心性格有些内向的**针织工**，正在织毛衣。***针织工****踹了一下刺绣工/考古学家。*]

17a. There is an **acrobat** with a very solid foundation and a knack for hard work, standing next to a juggler (5.5)/librarian (1.8), performing. ***Acrobat*** *picking up the ball.*

[有一位基本功相当扎实特别能吃苦的**杂技演员**，站在一名杂耍艺人/图书管理员旁边，正在表演节目。***杂技演员****抱起了球。*]

17b. There is an **acrobat** with a very solid foundation and a knack for hard work, performing. ***Acrobat*** *patting juggler/librarian.*

[有一位基本功相当扎实特别能吃苦的**杂技演员**，正在表演节目。***杂技演员****拍了一下杂耍艺人/图书管理员。*]

18a. There is a very angry and agitated **plaintiff**, standing next to a prosecutor (6.0)/groom (1.6), accusing the defendant of his actions. ***Plaintiff*** *opening the tie.*

[有一位相当愤怒相当激动的**原告**，站在一名起诉人/新郎旁边，正在控诉被告的行为。***原告****解开了领带。*]

18b. There is a very angry and agitated **plaintiff**, accusing the defendant of his actions. ***Plaintiff*** *hitting prosecutor/groom.*

[有一位相当愤怒相当激动的**原告**，正在控诉被告的行为。***原告****打了一下起诉人/新郎。*]

19a. There is a very creative and ingenious **baker**, standing next to a pastry chef (5.7)/producer(2.1), serving out a cake that had just come out of the oven. ***Baker*** *laying hand on the cake.*

[有一位特别有创造力特别心灵手巧的**面包师**，站在一名面点师/制片人旁边，正在端出刚出炉的蛋糕。***面包师****手按在了蛋糕上。*]

19b. There is a very creative and ingenious **baker**, serving out a cake that had just come out of the oven. ***Baker*** *pinching pastry chef/producer.*

[有一位特别有创造力特别心灵手巧的**面包师**，正在端出刚出炉的蛋糕。***面包师****掐了一下面点师/制片人。*]

20a. There is a very young and articulate **news anchor**, sitting next to a show host (5.9)/gardener (2.3), checking manuscripts. ***News anchor*** *pounding the desk.*

[有一位很年轻口才特别好的**新闻主播**，坐在一名节目主持人/园丁旁边，正在核对稿件。***新闻主播****拍了一下桌子。*]

20b. There is a very young and articulate **news anchor**, checking manuscripts. ***News anchor*** *patting show host/gardener.*

[有一位很年轻口才特别好的**新闻主播**，正在核对稿件。***新闻主播****拍了一下节目主持人/园丁。*]

21a. There is a very loving and caring **nurse**, standing next to a care worker (5.6)/poetess (2.4), holding bottles of medicine to infuse. ***Nurse*** *knocking over the medicine bottles.*

[有一位非常有爱心非常关爱病人的**护士**，站在一名护工/女诗人旁边，正在端着药瓶准备挂水。***护士****碰翻了药瓶。*]

21b. There is a very loving and caring **nurse**, holding bottles of medicine to infuse. ***Nurse*** *pushing care worker/poetess.*

[有一位非常有爱心非常关爱病人的**护士**，正在端着药瓶准备挂水。***护士****推了一下护工/女诗人。*]

22a. There is a **psychological counsellor** with quick reaction and sharp thinking, standing next to a psychologist (6.1)/editor (2.4), chatting with a patient. ***Psychological counsellor*** *knocking over the coffee cup.*

[有一位反应很快思维很敏锐的**心理咨询师**，坐在一名心理医生/编辑旁边，正在和病人聊天。***心理咨询师****碰翻了咖啡杯。*]

22b. There is a **psychological counsellor** with quick reaction and sharp thinking, chatting with a patient. ***Psychological counsellor*** *kicking psychologist/editor.*

[有一位反应很快思维很敏锐的**心理咨询师**，正在和病人聊天。***心理咨询师****踹了一下心理医生/编辑。*]

23a. There is a very skilled and patient **obstetrician**, standing next to a midwife (5.2)/drummer (2.1), informing the family about the new baby. ***Obstetrician*** *removing her doctor’s coat.*

[有一位医术很高明特别有耐心的**妇产科医生**，站在一名助产士/鼓手旁边，正在告知家属新生儿情况。***妇产科医生****脱掉了白大褂。*]

23b. There is a very skilled and patient **obstetrician**, informing the family about the new baby.***Obstetrician*** *touching midwife/drummer.*

[有一位医术很高明特别有耐心的**妇产科医生**，正在告知家属新生儿情况。***妇产科医生****碰了一下助产士/鼓手。*]

24a. There is a very down-and-out and poor **beggar**, squatting next to a homeless people (5.1)/skydiver (1.6), rummaging through a bin. ***Beggar*** *knocking over the bin.*

[有一位非常落魄非常可伶的**乞丐**，蹲在一名无家可归者/跳伞运动员旁边，正在垃圾桶前翻吃的。***乞丐****打翻了垃圾桶。*]

24b. There is a very down-and-out and poor **beggar**, rummaging through a bin. ***Beggar*** *touching homeless people/skydiver.*

[有一位非常落魄非常可伶的**乞丐**，正在垃圾桶前翻吃的。***乞丐****碰了一下无家可归者/跳伞运动员。*]

25a. There is a **tallyman** in a hat and overalls, standing next to a warehouse keeper (5.6)/designer (2.4), taking inventory. ***Tallyman*** *picking up the box.*

[有一位带着帽子穿着背带裤的**理货员**，站在一名仓库管理员/设计师旁边，正在清点物品。***理货员****搬起了箱子。*]

25b. There is a **tallyman** in a hat and overalls, taking inventory. ***Tallyman*** *kicking warehouse keeper/designer.*

[有一位带着帽子穿着背带裤的**理货员**，正在清点物品。***理货员****踢了一下仓库管理员/设计师。*]

26a. There is a **hostage** who is covered with his mouth and tightly bound, sitting next to a captive (5.3)/artist (2.4), thinking about how to escape. ***Hostage*** *hiding under the table.*

[有一位被捂着嘴绑的严严实实的**人质**，坐在一名俘虏/艺术家旁边，正在思考如何逃脱。***人质****躲在了桌子下面。*]

26b. There is a **hostage** who is covered with his mouth and tightly bound, thinking about how to escape. ***Hostage*** *stepping on captive/artist.*

[有一位被捂着嘴绑的严严实实的**人质**，正在思考如何逃脱。***人质****踩了一下俘虏/艺术家。*]

27a. There is a very excited **audience**, sitting next to a listener (5.6)/legal advisor (2.3), waiting for the show to begin. ***Audience*** *putting feet up in the chair.*

[有一位特别兴奋特别激动的**观众**，坐在一名听众/法律顾问旁边，正在等待演出开始。***观众****把脚翘到了椅子上。*]

27b. There is a very excited **audience**, waiting for the show to begin. ***Audience*** *patting listener/legal advisor.*

[有一位特别兴奋特别激动的**观众**，正在等待演出开始。***观众****拍了一下听众/法律顾问。*]

28a. There is a very penetrating **critic** with a unique perspective, standing next to a commentator (5.2)/waiter (2.4), reviewing a newspaper article. ***Critic*** *throwing the newspaper on the floor.*

[有一位相当犀利见解特别独到的**批评家**，站在一名评论员/服务员旁边，正在点评报纸上的一篇文章。***批评家****把报纸扔在了地上。*

28b. There is a very penetrating **critic** with a unique perspective, reviewing a newspaper article. ***Critic*** *kicking commentator/waiter.*

[有一位相当犀利见解特别独到的**批评家**，正在点评报纸上的一篇文章。***批评家****踹了一下评论员/服务员。*]

29a. There is a **secretary** in business attire who is very busy, standing next to an assistant (5.9)/chef (2.1), answering the phone. ***Secretary*** *dropping the phone on the floor.*

[有一位穿着职业装工作十分忙碌的**秘书**，站在一名助理/厨师旁边，正在接电话。***秘书****把电话扔在了地上。*]

29b. There is a **secretary** in business attire who is very busy, answering the phone. ***Secretary*** *pulling assistant/chef.*

[有一位穿着职业装工作十分忙碌的**秘书**，正在接电话。***秘书****拉了一下助理/厨师。*]

30a. There is a very talented and thoughtful **novelist**, sitting next to a playwright (5.3)/programmer (2.1), writing an article. ***Novelist*** *falling asleep on the desk.*

[有一位非常有才华非常有思想的**小说家**，坐在一名剧作家/程序员旁边，正在写文章。***小说家****趴在桌上睡着了。*]

30b. There is a very talented and thoughtful **novelist**, writing an article. ***Novelist*** *pushing playwright/programmer.*

[有一位非常有才华非常有思想的**小说家**，正在写文章。***小说家****推了一下剧作家/程序员。*]

31a. There is a very talented and good **singer**, standing next to a musician (5.3)/taxi driver (2.3), signing a song. ***Singer*** *dropping the microphone on the floor.*

[有一位特别有天赋歌唱的特别好听的**歌手**，站在一名音乐家/出租车司机旁边，正在演唱歌曲。***歌手****把话筒扔在了地上。*]

31b. There is a very talented and good **singer**, signing a song. ***Singer*** *patting musician/taxi driver.*

[有一位特别有天赋歌唱的特别好听的**歌手**，正在演唱歌曲。***歌手****拍了一下音乐家/出租车司机。*]

32a. There is a very creative **barber** with a very good attitude, standing next to a hairdresser

(6.5)/journalist (2.4), designing a hairstyle. ***Barber*** *dropping the scissors on the floor.*

[有一位特别有创造力态度非常好的**理发师**，站在一名发型师/记者旁边，正在设计发型。***理发师****把剪刀扔在了地上。*]

32b. There is a very creative **barber** with a very good attitude, designing a hairstyle. ***Barber*** *stepping on hairdresser/journalist.*

[有一位特别有创造力态度非常好的**理发师**，正在设计发型。***理发师****踩了一下发型师/记者。*]

33a. There is a very imaginative and artistic **painter**, standing next to an illustrator (5.3)/butcher (2.5), thinking about a drawing. ***Painter*** *dropping the tray on the floor.*

[有一位想象力特别丰富特别有文艺气息的**画家**，站在一名插画师/屠夫旁边，正在构思画作。***画家****把画盘扔在了地上。*]

33b. There is a very imaginative and artistic **painter**, thinking about a drawing. ***Painter*** *hitting illustrator/butcher.*

[有一位想象力特别丰富特别有文艺气息的**画家**，正在构思画作。***画家****打了一下插画师/屠夫。*]

34a. There is a very fair and courageous **judge**, standing next to a prosecutor (5.4)/cleaner (1.9), waiting for the court session. ***Judge*** *dropping the code on the floor.*

[有一位非常公正非常有魄力的**法官**，站在一名检察官/清洁工旁边，正在等待开庭。***法官****把法典掉在了地上。*]

34b. There is a very fair and courageous **judge**, waiting for the court session. ***Judge*** *stepping on prosecutor/cleaner.*

[有一位非常公正非常有魄力的**法官**，正在等待开庭。***法官****踩了一下检察官/清洁工。*]

35a. There is a bespectacled **doctor** with very rich clinical experiences, standing next to a pharmacist (5.3)/president (2.6), touring the ward. ***Doctor*** *dropping the medical record on the floor.*

[有一位戴着眼镜临床经验非常丰富的**医生**，站在一名药剂师/总统旁边，正在巡视病房。***医生****把病历本掉在了地上。*]

35b. There is a bespectacled **doctor** with very rich clinical experiences, touring the ward. ***Doctor*** *pinching pharmacist/president.*

[有一位戴着眼镜临床经验非常丰富的**医生**，正在巡视病房。***医生****掐了一下药剂师/总统。*]
